# Trichilemmal carcinoma treated by excision combined with photodynamic therapy: a case report and review of literature

**DOI:** 10.3389/fonc.2024.1436399

**Published:** 2024-11-22

**Authors:** Lingyu Qidiao, Yilin Liu, Danni Hu, Xingchi Tao, Chunli Yao

**Affiliations:** Department of Dermatology, Second Affiliated Hospital of Jilin University, Changchun, China

**Keywords:** trichilemmal carcinoma, photodynamic therapy, rare skin tumor, case report, review

## Abstract

Trichilemmal carcinoma is an extremely rare malignant cutaneous tumor derived from the outer root sheath of the hair follicles, which most commonly occurs in the sun-exposed areas of elderly individuals. This article introduces a case of trichilemmal carcinoma that occurred on the scalp of a 36-year-old male patient, the first case reported and treated with surgical excision combined with photodynamic therapy.

## 1 Introduction

Trichilemmal carcinoma (or tricholemmal carcinoma [TC]) is a rare malignant cutaneous appendage tumor derived from the outer root sheath of the hair follicles. It most frequently occurs in the sun-exposed areas of elderly individuals, typically affecting the head, face, neck, or upper extremities (1, 2). Its skin lesions often have atypical appearances, necessitating carefully differential diagnosis with other possible lesions (3). The treatment of choice is complete surgical excision (4). Other reported therapies include imiquimod cream (5%) (5), radiotherapy, and chemotherapy (6), whereas the use of photodynamic therapy (PDT) has not been documented in the literature. Herein, we present a case of trichilemmal carcinoma treated with surgical resection combined with PDT.

## 2 Case report

**Figure 1:**
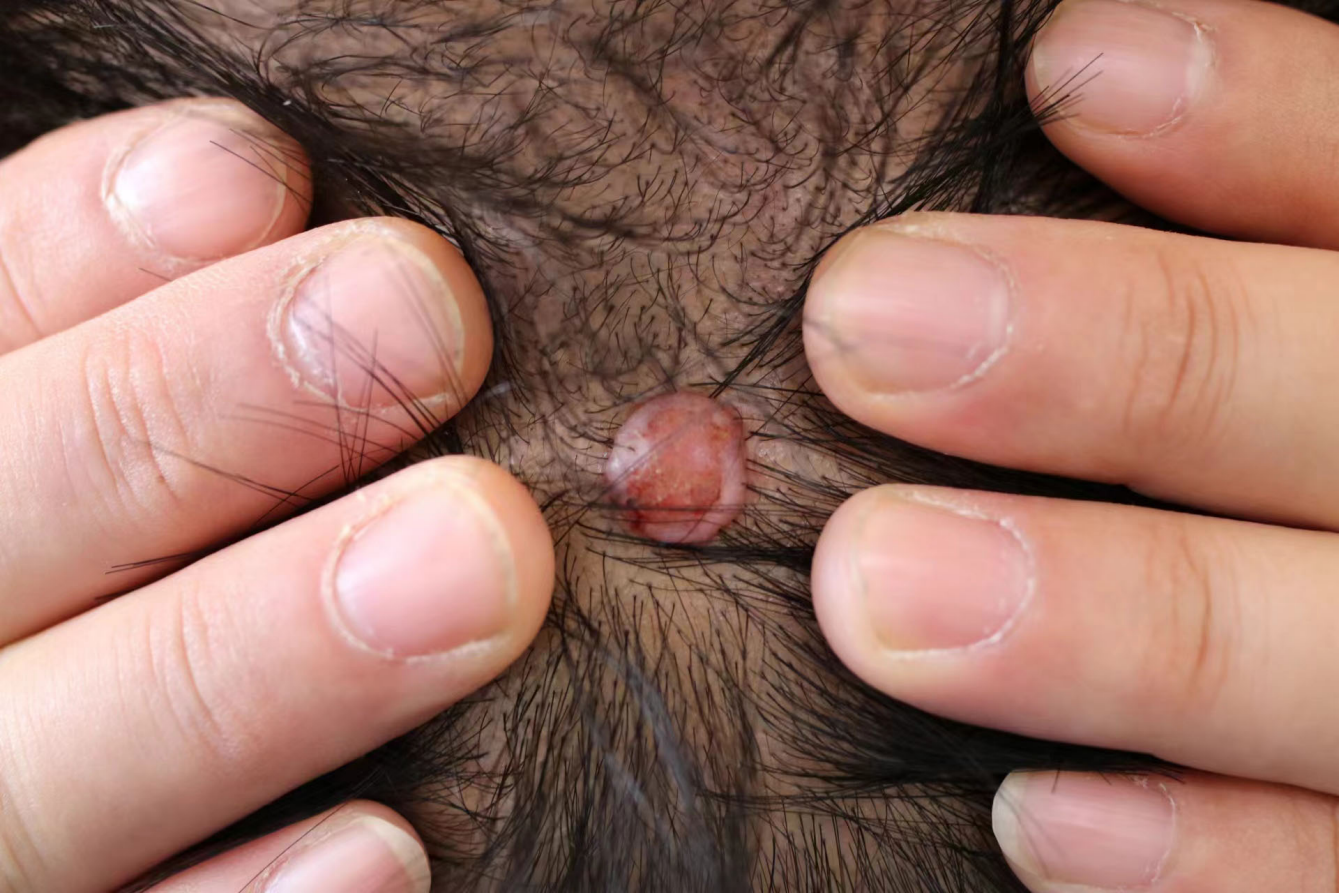
Clinical photograph before surgery.

In November 2023, the patient presented to our hospital with a reddish skin swelling on the top of the head that had been present for 10 months without an obvious cause. The patient complained of localized itching and swelling sensation, habitual scratching, and no prolonged sun exposure. He denied any relevant family history or hereditary diseases, as well as any history of surgery, trauma, or similar conditions. He had a smoking history of 10 years. Previously, the patient had been admitted to a local hospital and had undergone a pathologic examination on an outpatient basis, which revealed clear cell hidradenocarcinoma. Upon hospitalization at the Second Hospital of Jilin University, physical examination revealed a light red skin swelling on the top of the head, approximately 1.0 cm×1.0 cm, protruding from the skin surface, with a clear border and ulcerated surface (**Figure 1**). No enlarged lymph nodes were detected in the head and neck. The preliminary diagnosis was clear cell hidradenocarcinoma.

**Figure 2:**
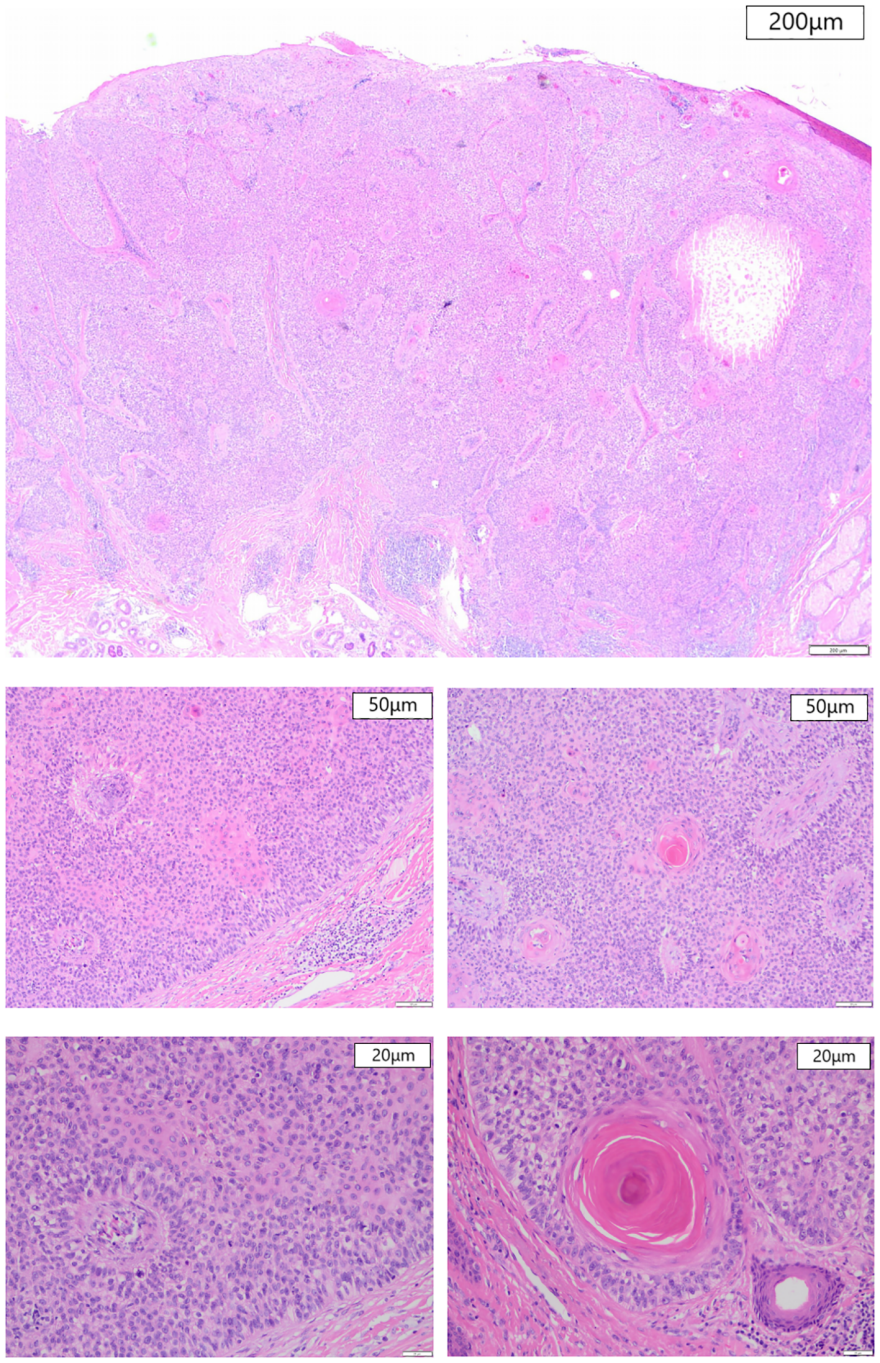
Histological section, HE staining. **(A)** At magnification 20× (200μm), a necrotic foci can be seen at the right side in large-sized tumors. **(B, C)** At magnification 100× (50μm), follicular keratinization and palisaded cells can be seen. **(D, E)** At magnification 200× (20μm), a clear cytoplasm containing atypical nuclei and mitotic figures is seen.

**Figure 3:**
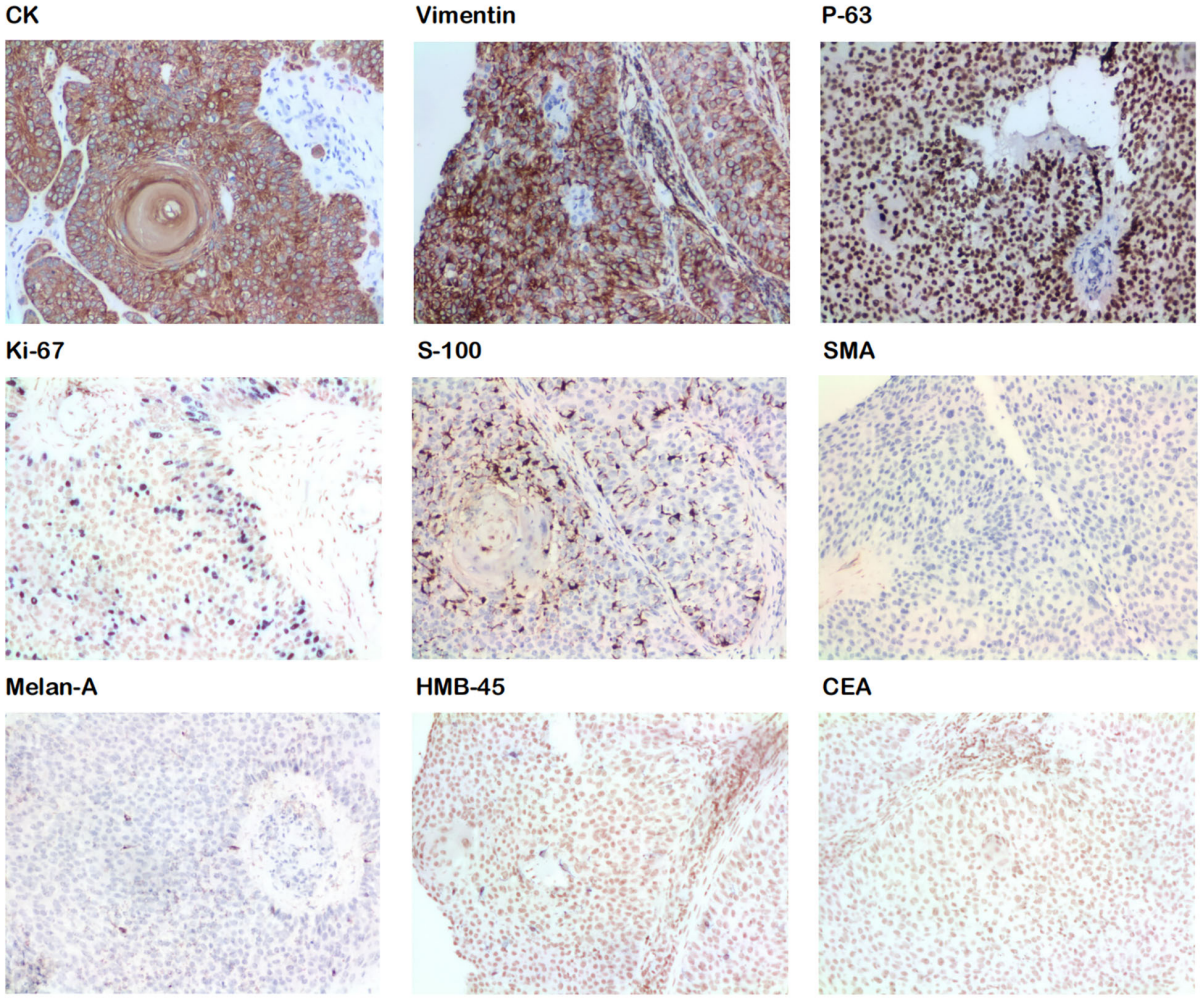
Immunohistochemical staining shows positive results for CK, vimentin, P-63, Ki-67, and S-100 and negative results for SMA, Melan A, HMB-45, and CEA.

**Figure 4:**
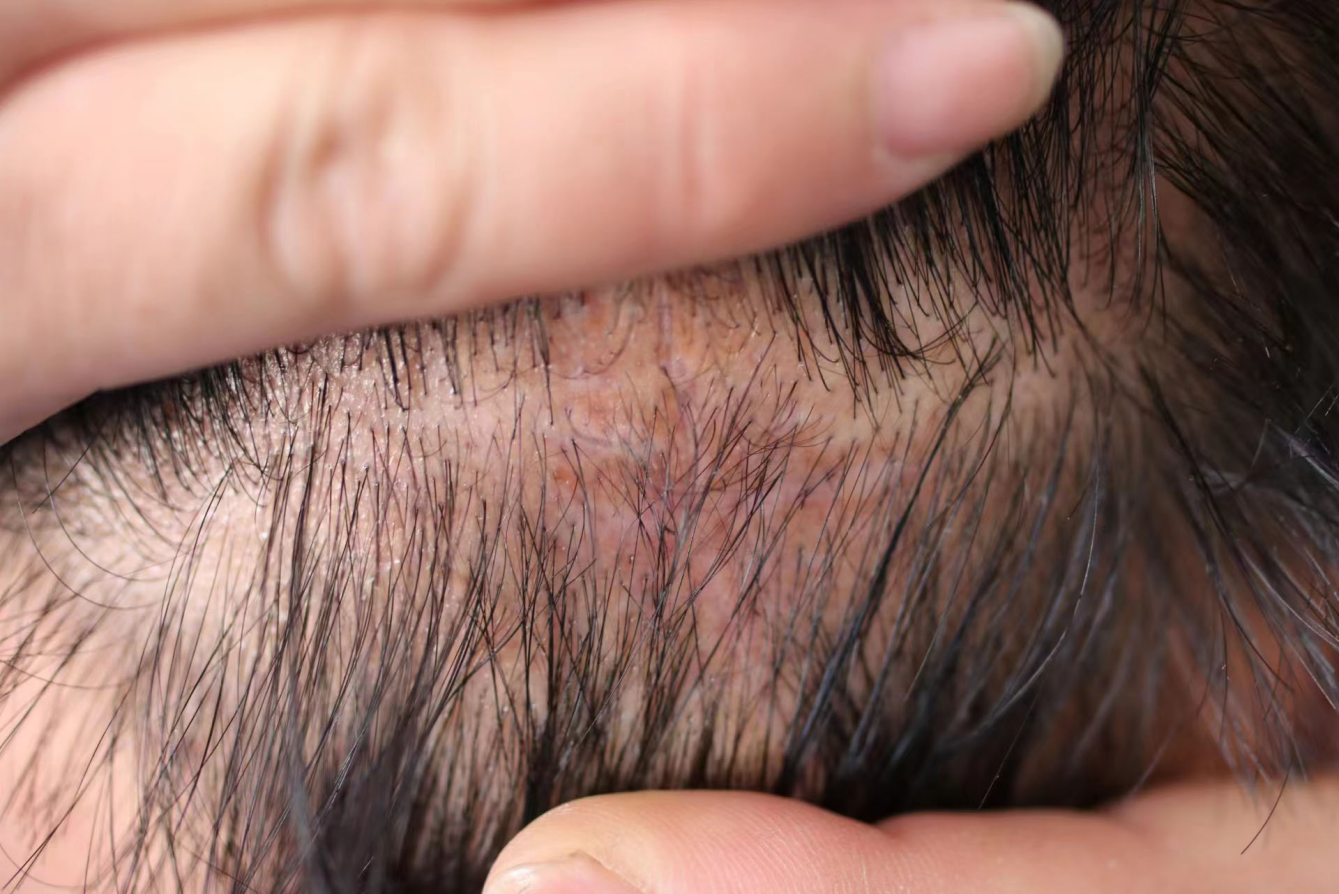
At the 6-month follow-up, no sign of recurrence or metastasis is noted.

After preoperative preparation, excision of the skin lesion was performed under local anesthesia, with a margin of 2.0 cm×1.0 cm and a depth extending to the subcutaneous tissue. Intraoperative rapid pathology results showed negative margins, and intraoperative photodynamic adjuvant therapy was administered. Postoperative pathological findings indicated a tumor derived from the hair follicles, with focal suspicious cell infiltration and evident mitotic figures, consistent with TC (**Figures 2**, **3**). The surgical margins were negative. Upon wound healing, additional two sessions of photodynamic adjuvant therapy were administered. After a 6-month follow-up, there were no abnormalities detected in the ultrasound examination of cervical lymph nodes, and no signs of local recurrence (**Figure 4**). Both the doctor and the patient are satisfied with the treatment effect. No unexpected events or adverse events occurred during the treatment and follow-up process.

## 3 Discussion and conclusions

TC is an extremely rare, malignant skin tumor originating from the outer root sheath of the hair follicle. It was first described in 1976 by J. T. Headington (6), and since then approximately 231 cases have been reported (2). It typically occurs in elderly individuals on sun-exposed areas such as the head, neck, arms, and hands (3, 7, 8). The pathogenesis of TC remains unclear, but potential risk factors include advanced age, ultraviolet light exposure, ionizing radiation, a history of trauma, cicatrix, hereditary diseases, and immunosuppression in organ transplant recipients (3, 6, 9). However, in the present case, the patient lacked these risk factors. Smoking history may need consideration, as it is unclear whether smoking is a significant risk factor for TC, its association with TC development requires further study.

The appearance of TC skin lesions is non-specific, often presenting as single prominent swellings that are red, brown, or skin-colored, which may sometimes be accompanied by redness, bleeding, and ulceration. These lesions resemble basal cell carcinoma (BCC), squamous cell carcinoma (SCC), keratoacanthoma, or other follicle-derived skin lumps, leading to misdiagnosis (10–12). Therefore, diagnosis mainly relies on histopathology. In this case, preliminary diagnosis was made based on the appearance of skin lesions and third-party pathological examination results. Both being rare tumors of skin appendages, TC and hidradenocarcinoma share similar pathological manifestations. Also, both of them have nonspecific clinical presentations, posing a challenge for the differential diagnosis between these two diseases. The final diagnosis relied on the pathological examination results from our hospital after complete excision, which exhibited the characteristic follicular keratinization of TC. Also, they did not align with the features of hidradenocarcinoma, such as non-invasion of the epidermis, presence of eosinophilic granulocyte components, and tendency to invade surrounding tissues (13). Additionally, the immunohistochemical results, CK (+) and CEA (–) were inconsistent with the characteristics of hidradenocarcinoma reported in the literature (14).

Currently, no uniform pathological diagnostic standard has been established for TC. Certain histopathological features of TC include the following: 1) Tumor cells with clear cytoplasm, containing an increased number of atypical nuclei and mitotic figures (3, 6, 7); 2) Positive staining for periodic acid-Schiff (PAS), sensitive to diastase (1), and negative to mucin (12); 3) Lobular or trabecular proliferation centered around pilosebaceous structures (3, 6, 15–17); 4) Palisaded cells surrounding tumor tissues (2, 7, 12); 5) Trichilemmal keratinization (1, 7); and 6) Mostly positive immunostaining for Pan CK, CK15, Ki-67, p63, p53, and CK1 but negative for S-100, CEA, HMB-45, Vimentin, Melan A, and SMA. EMA was widely adopted but it’s not suitable as diagnostic criteria, for its positive and negative results occur with the same frequency. Other pathological indicators have limited data (2).

Dermoscopy is essential for the diagnosis of most skin cancers. Arslan et al. (7) reported a case of TC initially misdiagnosed as BCC due to similar dermoscopic features such as blood vessels of varying thickness and unstructured yellow and white areas. Their study concluded that it is difficult to distinguish TC from other non-melanoma skin cancers by dermoscopic manifestations, as previous studies have reported arborizing blood vessels, yellow areas, and lobular structures in both follicle-derived tumors and BCCs. Sun et al. (2) reported a case of TC with asymmetric lobular nodules with crystalline chrysalis-like structures, which was preliminarily diagnosed as BCC. So far, no studies have described the dermatoscopic morphology of TC in addition to the above reports, warranting further study. Based on these findings, it can be assumed that dermoscopy may not be a reliable tool for diagnosing TC.

Histologic features suggest that TC is a moderate-to-severe malignancy, but it typically follows a indolent process and is not prone to recurrence or metastasis (4). Simple excision, which is currently the preferred treatment for non-metastatic TC, is highly effective in most cases when the tumor is completely excised with clear margins (12) and has a local recurrence rate of 7.55% (2). Mohs micrographic surgery (MMS) is a more desirable option as it allows for better preservation of healthy tissue while removing as much of the tumor tissue as possible. However, the postoperative recurrence rate with MMS is not significantly different compared with that with simple excision (2), which may be due to limited data. In addition, the economic costs of MMS, its requirements for personnel and equipment, as well as its relatively complicate procedures, also pose constraints on its practical application.

Other interventions can be used in patients who are unsuitable or decline to undergo surgery and as adjunctive therapy for surgical patients. One reported case successfully treated a 90-year-old woman with the application of 5% imiquimod cream (5). Another distant metastasis case underwent pembrolizumab treatment, achieved good response and disease control, after ineffective surgeries, radiation and chemotherapy (18).However, immunotherapy must be adapted to each patient, as they can react differently to such drugs, with various side effects (19). There is no standard radiotherapy plan or chemotherapy regimen for TC. According to the previous reports (20), A 27-year-old TC patient successfully relieved his 5-cm lesion on the neck, who recieved chemotherapy and radiotherapy after excision. Another research (2) recommended that, for nodules larger than 5 cm in diameter, radiotherapy or chemotherapy can be considered. Yi et al. (21) suggested that chemotherapy regimens designed for advanced SCC can be used as an intervention for metastatic TC because of their similarities in cytology. The effects of chemotherapy on prolonging the survival of patients with metastatic cancer, reducing tumor burden, and improving quality of life are worth considering. However, achieving a cure is still challenging. For lesions that have not yet recurred or metastasized, larger tumor size and longer disease duration increase the risk of recurrence (2). Similar to other skin tumors, if the pathological examination reports perineural invasion, perineural chronic inflammation, or lymphovascular invasion, it may also indicate a poor prognosis (22), thereby potentially necessitating radiotherapy or chemotherapy.

In this case, the patient underwent intra- and postoperative photodynamic adjuvant therapy using the photosensitizer 5-aminolevulinic acid (ALA), which is a light-activated compound that produces reactive oxygen species, leading to the destruction of tumor cells and blood vessels while stimulating the immune system, then selectively eliminate tumor cells (23), ensure negative margins and minimize the risk of recurrence. For our patient with TC, PDT has the following advantages compared with chemical therapy and radiotherapy: 1) good selectivity and slight toxic side effects: almost no damage to normal tissues and no influence on hematopoietic function; 2) cost effectiveness: the consumption of photodynamic therapy is significantly lower than that of postoperative radiotherapy, which reduces the patient’s economic burden; and 3) preservation of appearance and function: PDT has a better cosmetic effect on head and face skin tumors. On the premise of achieving the therapeutic purpose, it can utmost ensure the integrity of appearance and function, which is also conducive to reducing the psychological burden of patients. The patient underwent a total of three PDT sessions intraoperatively, one week postoperatively, and two weeks postoperatively. The preparation used was a 20% solution of 5-aminolevulinic acid hydrochloride. The solution was applied to the surgical site, black-covered, and left for 3 hours before being irradiated with 630nm ± 10nm red light for 20 minutes at a power density of 60mW/cm² (24).

PDT is indicated for a wide range of non-melanoma skin cancers, including actinic keratosis, Bowen’s disease (SCC *in situ*), and BCC. However, it has the following limitations: 1) it can be painful; 2) its effectiveness decreases with increasing tumor thickness; and 3) it is not applicable to morphoeic BCC, infiltrative BCC, pigmented BCC, and invasive SCC and other tumor types (25). This is because thicker, denser tumors, those invading deeper tissues, or those with more melanin pigment may prevent the light-activated compound from reaching all tumor cells or the light from penetrating deeply enough, thereby hindering the effectiveness of PDT. However, in our case, the intraoperative combined application of PDT circumvented the limitations of tumor thickness and light penetration, and its therapeutic significance lies in ensuring negative margins, eliminating possible residual tumor cells, and minimizing the risk of recurrence. No local recurrence or metastasis was observed during the 6-month postoperative follow-up, but the long-term efficacy requires monitoring.

We recommend follow-up visits for TC patients at 3, 6, and 12 months after discharge, then annually, emphasizing sun protection during this period. Long-term follow-up with thorough examinations is necessary due to the limited knowledge and lack of established consensus on TC management.

To summarize the characteristics of this patient with TC: 1) The cause of the disease is unknown. The history did not involve any known risk factors. 2) It was misdiagnosed as clear cell hidroadenocarcinoma on admission to the hospital. This suggests the significance of differential diagnosis of this disease, and more studies are needed to summarize the findings of more typical clinical and pathological features to establish clearer diagnostic criteria. 3) This is the first reported case of TC treated with surgery combined with photodynamic adjuvant therapy. This treatment can theoretically achieve a better therapeutic effect and lower side effects, reduce treatment cost, and better cosmetic results.

## Data Availability

The original contributions presented in the study are included in the article/supplementary material. Further inquiries can be directed to the corresponding author/s.
